# Capture-Recapture Estimators in Epidemiology with Applications to Pertussis and Pneumococcal Invasive Disease Surveillance

**DOI:** 10.1371/journal.pone.0159832

**Published:** 2016-08-16

**Authors:** Toon Braeye, Jan Verheagen, Annick Mignon, Wim Flipse, Denis Pierard, Kris Huygen, Carole Schirvel, Niel Hens

**Affiliations:** 1 Department Epidemiology of infectious diseases, Epidemiology, Scientific Institute of Public Health, Brussels, Belgium; 2 Department of Clinical Microbiology, University Clinic Leuven, Leuven, Belgium; 3 Medical Affairs, Pfizer Vaccines, Brussels, Belgium; 4 Infectious Disease Control, Flemish Agency for Care and Health, Brussels, Belgium; 5 Institute of Medical Microbiology, University Hospital of Brussels, Brussels, Belgium; 6 Department immunology, Communicable and Infectious Diseases, Scientific Institute of Public Health, Brussels, Belgium; 7 Cellule de surveillance des maladies infectieuses, Direction générale de la santé, Brussels, Belgium; 8 Interuniversity Institute for Biostatistics and statistical Bioinformatics, Hasselt University, Hasselt, Belgium; 9 Centre for Health Economic Research and Modelling Infectious Diseases (CHERMID), Vaccine & Infectious Disease Institute (WHO Collaborating Centre), University of Antwerp, Wilrijk, Belgium; 10 Epidemiology and social medicine (ESOC), University of Antwerp, Wilrijk, Belgium; Ohio State University College of Medicine, UNITED STATES

## Abstract

**Introduction:**

Surveillance networks are often not exhaustive nor completely complementary. In such situations, capture-recapture methods can be used for incidence estimation. The choice of estimator and their robustness with respect to the homogeneity and independence assumptions are however not well documented.

**Methods:**

We investigated the performance of five different capture-recapture estimators in a simulation study. Eight different scenarios were used to detect and combine case-information. The scenarios increasingly violated assumptions of independence of samples and homogeneity of detection probabilities. Belgian datasets on invasive pneumococcal disease (IPD) and pertussis provided motivating examples.

**Results:**

No estimator was unbiased in all scenarios. Performance of the parametric estimators depended on how much of the dependency and heterogeneity were correctly modelled. Model building was limited by parameter estimability, availability of additional information (e.g. covariates) and the possibilities inherent to the method. In the most complex scenario, methods that allowed for detection probabilities conditional on previous detections estimated the total population size within a 20–30% error-range. Parametric estimators remained stable if individual data sources lost up to 50% of their data. The investigated non-parametric methods were more susceptible to data loss and their performance was linked to the dependence between samples; overestimating in scenarios with little dependence, underestimating in others. Issues with parameter estimability made it impossible to model all suggested relations between samples for the IPD and pertussis datasets. For IPD, the estimates for the Belgian incidence for cases aged 50 years and older ranged from 44 to58/100,000 in 2010. The estimates for pertussis (all ages, Belgium, 2014) ranged from 24.2 to30.8/100,000.

**Conclusion:**

We encourage the use of capture-recapture methods, but epidemiologists should preferably include datasets for which the underlying dependency structure is not too complex, a priori investigate this structure, compensate for it within the model and interpret the results with the remaining unmodelled heterogeneity in mind.

## Introduction

When epidemiologists report disease incidence estimates and trends in disease incidence from surveillance data, the proportion of unreported and undetected cases is an important consideration. The unknown sensitivity of a non-exhaustive surveillance system is one of the main reason why capture-recapture methods have been explored in epidemiology [1]. Capture–recapture methods allow for the estimation of an unknown population size by using two or more samples from that population. Approaches with only one sample, in which the number of occurrences of cases are modelled, have also been developed [2]. The methodology has largely been developed in the field of ecology for abundance estimation [3]. In its most classical form an estimate of the size of a species’ population is calculated after capturing, tagging and recapturing animals. Whether or not an animal is tagged at recapture, is recorded as its capture-recapture history. If an identifying variable can be recorded on a list at every capture occasion, tagging is unnecessary. The capture-recapture histories become apparent by merging the lists. The methods can directly be translated to epidemiology. Surveillance systems serve as capture occasions and the probability of detection is the capture probability.

Assumptions are necessary for an unbiased estimation of the total number of cases [4]. Method development has focussed on relaxing these assumptions by offering different ways to model, part of, the dependence between sources or by finding a lower bound for the total population size [5]. Dependence is the existence of a direct causal effect on inclusion in a sample if a case is also included in another sample [1]. Multiple factors can be responsible for dependence between samples and dependence can be further divided into direct and indirect dependence. Dependence can be caused directly by the design of surveillance systems and referrals between systems or it can be an indirect result of heterogeneous detection probabilities [2,6]. The direct and indirect dependence between samples form, what we call, the underlying dependency structure of the final dataset of capture histories. An overview of terminology is given in S1 Appendix.

Though this underlying dependency structure is only latent in the data, it results in a biased capture-recapture estimator if incompletely accounted for in the model. The size of this bias is unknown as the quantity of the remaining heterogeneity and the effect of necessarily neglecting remaining heterogeneity are specific to a certain estimator and situation [7,8]. Since the underlying dependency structure is assumed to be complex when readily available epidemiological datasets are used, previous reviews have warned against the use of capture-recapture methods in epidemiology and called it an “act of faith” [5,9–12]. The actual performance of different capture-recapture methods and their applicability in an epidemiological context is however incompletely investigated.

The capture-recapture methods that were first developed show a direct relation between dependence and bias. As the dependence between sources increases, the bias of the Lincoln-Petersen estimator and the Chapman estimator, will increase; the relative bias of the estimators is a function of the odds ratio [13,14]. This has been discussed in an epidemiological context in the late 1960s and early 1970s by Wittes [15]. She suggested to combine highly dependent lists to remedy source dependence. As a sensitivity test she suggested to calculate estimates after a source was left out and compare abundance estimates. Fienberg and Bishop suggested to model the dependence between sources and introduced the, now still commonly used, log-linear Poisson models [16]. In the 1990s methods became available in which individual covariates and mixtures could be used to model detection probabilities [17–21]. A review on the use of capture-recapture methods in epidemiology is given by Schwarz and Seber and Hook and Regal [22,23]. An overview of estimators is given by Chao [24].

Our aim was to investigate the performance of capture-recapture estimators in a context of readily available surveillance data. We simulated data, captured it with three surveillance systems under different, increasingly complex, scenarios and investigated the bias of five estimators previously used in epidemiological capture-recapture studies. As more sources of dependence were included, the scenario resulted in a more complex underlying dependency structure. Possible sources of dependence included; referrals, covariate-dependent sampling and spatial heterogeneity of detection probabilities. We tried to include dependence as it would occur in an actual surveillance setting.

In addition to the simulation study, we estimated the incidence of Belgian Invasive Pneumococcal Disease (IPD) cases and pertussis cases. IPD and pertussis were chosen because of their clinical relevance. For both diseases, questions about vaccine efficiency warrant close surveillance and additional research [25–27]. Unlike in the simulation study the incidence-estimates could not be validated, but the applicability of the methods could be investigated and the range of the estimates could be evaluated. The feasibility and added value of guiding model selection and model building by the advice and opinions of experts and coordinators of the surveillance systems were also evaluated.

## Methods

### Description of the simulation study

In the simulation study we created 500 datasets with 5000 cases each. The birthdate and gender of the cases were sampled from the birthdate- and gender-distributions of real IPD cases found in sentinel surveillance. The selection of postcodes was weighted by the number of residents aged 50 years and more registered at these postcode. Eight different scenarios determined how and which cases were detected by three different surveillance systems. In the remainder of the text these systems will be called the sentinel, National Reference Center (NRC) and hospital sample. The scenarios are given below.

Random sampling (*M*_*t*_): Cases were equally likely detected. The number of cases detected in a sample was determined by the number of participating detectors (labs, hospitals) over the total number of detectors ($c_{sentinel}\sim \frac{labs\;participating\;in\;the\;sentinel\;surveillance}{all\;active\;labs}$).

Covariate-dependent sampling (*M*_*th*.*age*_): The probability of being detected by one of the three samples increased (*probability weights* = *age*(*days*)^2.5) with the age of the case. A 100 year old case was 2^2.5 times more likely to be detected than a 50 year old case. The size of the samples is determined as in random sampling ($c_{sentinel|age}\sim \frac{labs\;participating\;in\;the\;sentinel\;surveillance}{all\;active\;labs}|age$).

Spatial heterogeneity (*M*_*th*.*spat*_): The probability of being detected is determined by the location of the case, relative to the location of the detectors. A case is detected by one of the three nearest detectors. This detector is not necessarily participating in a specific surveillance system. The subset of detectors that participated in a surveillance network was based on the IPD dataset. The capture probability is equal for the three nearest detectors. The size of the samples is determined as in random sampling ($c_{sentinel|location}\sim p(\frac{labs\;participating\;in\;the\;sentinel\;surveillance}{all\;active\;labs}|location)$).

Referrals (*M*_*tb*.*ref*_): After random sampling, cases were referred from one sample to the other; 30% of the sentinel lab cases were referred to the NRC, 40% of the hospital cases were referred to the NRC $(c_{NRC|captured\;in\;sentinel\;sample}\sim \frac{labs\;participating\;in\;the\;NRC\;surveillance}{all\;active\;labs}+0.3(\frac{labs\;not\;participating\;in\;the\;NRC\;surveillance}{all\;active\;labs})).$

Every case was given a unique ID-variable during simulation. Surveillance systems listed captured cases after which the lists were merged by the ID-variable. For every simulation run of every scenario there was a final datasets, consisting of case characteristics (ID, postcode, birthdate, gender, age) and capture characteristics (detecting lab, detecting hospital, distance to detecting lab, distance to detecting hospital, detection date and detection history).

In the above scenarios only *M*_*t*_ (random sampling) has no source of dependence. The scenarios that introduced dependence were compared to the scenario of random sampling to demonstrate the effect of the dependence. The effect of sampling by age was illustrated by comparing the densities of the capture probability in the covariate-dependent scenario (*M*_*th*.*age*_) and the random scenario (*M*_*t*_). The spatial heterogeneity was illustrated with a kernel-smoothed relative risk function as computed by the R-package “sparr” [28]. The relative risk represents the ratio of detection probabilities (*M*_*th*.*spat*_/*M*_*t*_). The effect of referrals was illustrated by calculating the odds ratio for detection in one sample by detection in another sample. Additionally we constructed four more scenarios in which these sources of heterogeneity were combined. Three scenarios (*M*_*th*.*age*.*spat*_, *M*_*thb*.*age*.*ref*_, *M*_*thb*.*spat*.*ref*_)had two and one scenario (*M*_*thb*.*age*.*spat*.*ref*_) had three sources of heterogeneity.

### Description of the Belgian datasets on infectious diseases

Both the IPD and pertussis population were estimated by three-sample studies. The datasets were matched by a set of case characteristics (postcode, gender, birthdate) since no unique case identifier was present. If cases with identical identifiers were detected more than 90 days apart, they were treated as unique cases. The properties of the matching algorithm and additional sensitivity analysis were described in S1 Appendix. The data was collected under ethical approval or legislation. The hospital-based pneumococcal study was approved by the KULeuven ethical committee. The National Reference Centers is legislated by KB 09/02/2011. The mandatory notification in Flanders is legislated by the ‘preventiedecreet 21/11/2003’. The mandatory notification in Wallonia is legislated by ‘contrôle de la sécurité sanitaire 1/07/1998’. For the sentinel laboratory surveillance a statement has been submitted to the Belgian privacy commission.

#### Hospital-based IPD study

In Belgium, a hospital-based epidemiological study of IPD in adults was conducted between 2009 and 2011 [29]. The study was coordinated by a public-private partnership. Adults hospitalised with microbiologically confirmed IPD were eligible for inclusion. We limited the dataset to data on adults aged ≥50 years and only considered data collected between 1 July 2009 and 30 June 2011.

#### National reference centres (NRC)

The NRC analysed isolates they collected themselves and isolates they received from Belgian laboratories. Isolates were send to the NRC on a voluntary, but recommended, basis. The goals of an NRC were: confirmation and additional strain characterization (sero- and genotyping) and determining antibiotic resistance [30]. The NRC for *B*. *pertussis* were the laboratory at the University Hospital of Brussels and the Institute of Public Health. A titer of IgG antibodies against pertussis toxin (PT) >125 IU/ml, positive PCR or culture were considered as reflecting probable acute pertussis infection. The NRC dataset consisted of Belgian cases with samples collected in 2014.

The laboratory at the University Hospital of Leuven is the NRC for invasive isolates of *Streptococcus pneumoniae* bacteria. The IPD dataset was limited to those isolates obtained from normally sterile sites, in adults aged 50 years or older, between 1 July 2009 and 30 June 2011.

#### Sentinel laboratories network

*Streptococcus pneumoniae* and *Bordetella pertussis* were two of the 36 pathogens for which surveillance was organised through a sentinel network of laboratories [31]. The surveillance started in 1983 and consisted of both hospital laboratories and private laboratories. The network was coordinated by the Institute of Public Health. The IPD dataset was limited to those isolates obtained from normally sterile sites, in adults aged 50 years or older, between 1 July 2009 and 30 June 2011. The pertussis dataset consisted of all cases detected in 2014.

#### Mandatory notification

Notification of confirmed pertussis cases was mandatory in all three Belgian regions. Notification was coordinated by the regional public health agencies. Physicians and laboratories were obliged to notify cases, but notification was suspected to be incomplete. The pertussis dataset consisted of all cases notified in 2014.

### Estimators

Five different methods were used in the simulation study. The same methods were used for the estimation of the pertussis and IPD population. The methods have been used previously in epidemiological capture-recapture studies. We selected loglinear modelling, (conditional) multinomial likelihood, the non-parametric estimators; Burnham’s jackknife and Chao’s sample coverage and direct modelling of the underlying dependency structure with the Bayesian software WinBUGS (**Table 1**). A short description of the used estimators is given in S1 Appendix.

**Table 1:** Overview of the different estimators, their framework, reference to the literature, the software used and model selection by two approaches (1. Goodness of fit, 2. Assumptions on underlying dependency structure).

Framework	Method	Literature	Software	Model selection
Likelihood	Loglinear	Fienberg et al. [16]	R—Rcapture	1. Lowest AIC 2. Interaction terms ~ underlying structure
	Multinomial	Huggins et al., Alho et al. [18,19]	Mark, R—Rmark	1. Lowest AIC 2. Design matrix ~ underlying structure
Non-parametric	Sample Coverage	Chao et al. [33]	R—SPECIES	1. No model selection
	Jackknife	Burnham et al. [34]	R—SPECIES	1. No model selection
Bayesian	Bayesian	Jones et al. [6]	R—R2WinBUGS Winbugs	2. Directly modelled ~ underlying structure

#### Model selection and model building

We took two different approaches to select or build a model. With the first approach, assumptions on the underlying dependency structure were unnecessary; model selection was based on the goodness of fit. We selected the model with the lowest average AIC over the simulation runs by comparing boxplots. With the second approach, we tried to build or select the model that approximated the underlying dependency structure closest. The assumed underlying dependency structure was based on expert opinions for the real datasets and study design for the simulation study. Both approaches were possible with the multinomial and log-linear estimator. The two non-parametric estimators, for which there was no model selection, were reported together with the models selected by AIC (Table 2). The underlying dependency structure was approximated within log-linear models by adding interaction coefficients to those samples that were assumed dependent. Within multinomial models the design matrix was manually defined, so detection probabilities conditional on previous detection could be estimated. Finally within the Bayesian models the approach suggested by Jones et al. in which interactions and referrals are directly modelled, was taken [6]. Only the models where variance components could be estimated, were considered. For the Bayesian models convergence was assessed by the BGR-diagnostic, trace plots and the MC error [32].

**Table 2:** Models selected by AIC. For log-linear models a selection was made out of all hierarchical models. For the multinomial models a selection was made out of all possible models, including those with probabilities conditional on covariates and mixtures. *p*_1_ = p(sentinel), *p*_2_ = p(hospital), *p*_3_ = p(NRC), distance in meters, age in days. (*) parameters for the age and distance covariates were not estimable.

Scenario	Log-linear	Multinomial Likelihood
*M*_*t*_	∼ *p*_1_,*p*_2_,*p*_3_	∼ *p*_1_,*p*_2_,*p*_3_
*M*_*tb*.*ref*_	∼ *p*_1_,*p*_2_,*p*_3_, *p*_1_ * *p*_2_, *p*_2_ * *p*_3_, *p*_1_ * *p*_3_	∼ *p*_1_,*p*_2_,*p*_3_
*M*_*th*.*spat*_	∼ *p*_1_,*p*_2_,*p*_3_, *p*_1_ * *p*_2_, *p*_2_ * *p*_3_, *p*_1_ * *p*_3_	∼ *p*_1_,*p*_2_,*p*_3_, *distance*
*M*_*th*.*age*_	∼ *p*_1_,*p*_2_,*p*_3_, *p*_1_ * *p*_2_, *p*_2_ * *p*_3_, *p*_1_ * *p*_3_	∼ *p*_1_,*p*_2_,*p*_3_, *age*
*M*_*thb*.*spat*.*ref*_	∼ *p*_1_,*p*_2_,*p*_3_, *p*_1_ * *p*_2_, *p*_2_ * *p*_3_, *p*_1_ * *p*_3_	∼ *p*_1_,*p*_2_,*p*_3_, *distance*
*M*_*thb*.*age*.*ref*_	∼ *p*_1_,*p*_2_,*p*_3_, *p*_1_ * *p*_2_, *p*_2_ * *p*_3_, *p*_1_ * *p*_3_	∼ *p*_1_,*p*_2_,*p*_3_, *age*
*M*_*th*.*age*.*spat*_	∼ *p*_1_,*p*_2_,*p*_3_, *p*_1_ * *p*_2_, *p*_2_ * *p*_3_, *p*_1_ * *p*_3_	∼ *p*_1_,*p*_2_,*p*_3_(*)
*M*_*thb*.*age*.*spat*.*ref*_	∼ *p*_1_,*p*_2_,*p*_3_, *p*_1_ * *p*_2_, *p*_2_ * *p*_3_, *p*_1_ * *p*_3_	∼ *p*_1_,*p*_2_,*p*_3_, *distance*, *age*

### Analysis of the simulated data

Seven estimators from the combination of methods and model selection techniques (Table 2) were obtained for every simulation in each scenario. Four estimators were calculated without assumptions on the underlying dependency structure (first approach), for the other three estimators assumptions on the underlying dependency structure were necessary (second approach).

#### Comparison of estimators

We presented boxplots of the estimates of the total population size per estimator and per scenario. We compared the estimates to the actual population size of 5000 and to the average of unique cases per scenario over the simulation runs (unique cases-estimator). The relative bias was calculated as;

$$\frac{f_{0}-\hat{f_{0}}}{f_{0}}$$

With f_0_ = 5000 and $\hat{f_{0}}=$ population size estimate. The root of the mean of the squared relative bias was presented by method. (Boxplots of the relative bias are presented in S1 Appendix.)

#### The effect of randomly losing data on the estimators

To assess the effect of scarcity of data, 100 additional datasets were generated under the *M*_*t*_- and *M*_*thb*.*covar*.*spat*.*ref*_-scenario. The samples that constituted these datasets subsequently lost one percent of their cases. The total population size was estimated from the increasingly smaller datasets by log-linear modelling, the multinomial likelihood, Chao’s sample coverage and Bayesian models. The estimates were plotted over the loss of data and we analysed the trend for each method with a linear regression model. To investigate the stability of the estimation, the absolute value of the residuals of each model were plotted over the loss of data.

### Analysis of the datasets on IPD and pertussis

We describe the available data and the overlap between sources by Venn diagrams. The total population size of IPD and pertussis cases was then estimated by the five estimators, using both approaches. Assumptions on the dependency structure were suggested by the data owners. The presented confidence intervals are 95% confidence intervals (CI), as computed by the software packages (**Table 1**). Statistical significance was set at a significance level of 0.05 or smaller.

## Results of the simulation study

### Description of the simulated datasets

#### Effect of covariate-dependent heterogeneity (*M*_*th*.*age*_, *M*_*th*.*age*.*spat*_, *M*_*thb*.*age*.*ref*_, *M*_*thb*.*age*.*spat*.*ref*_)

**Fig 1:**
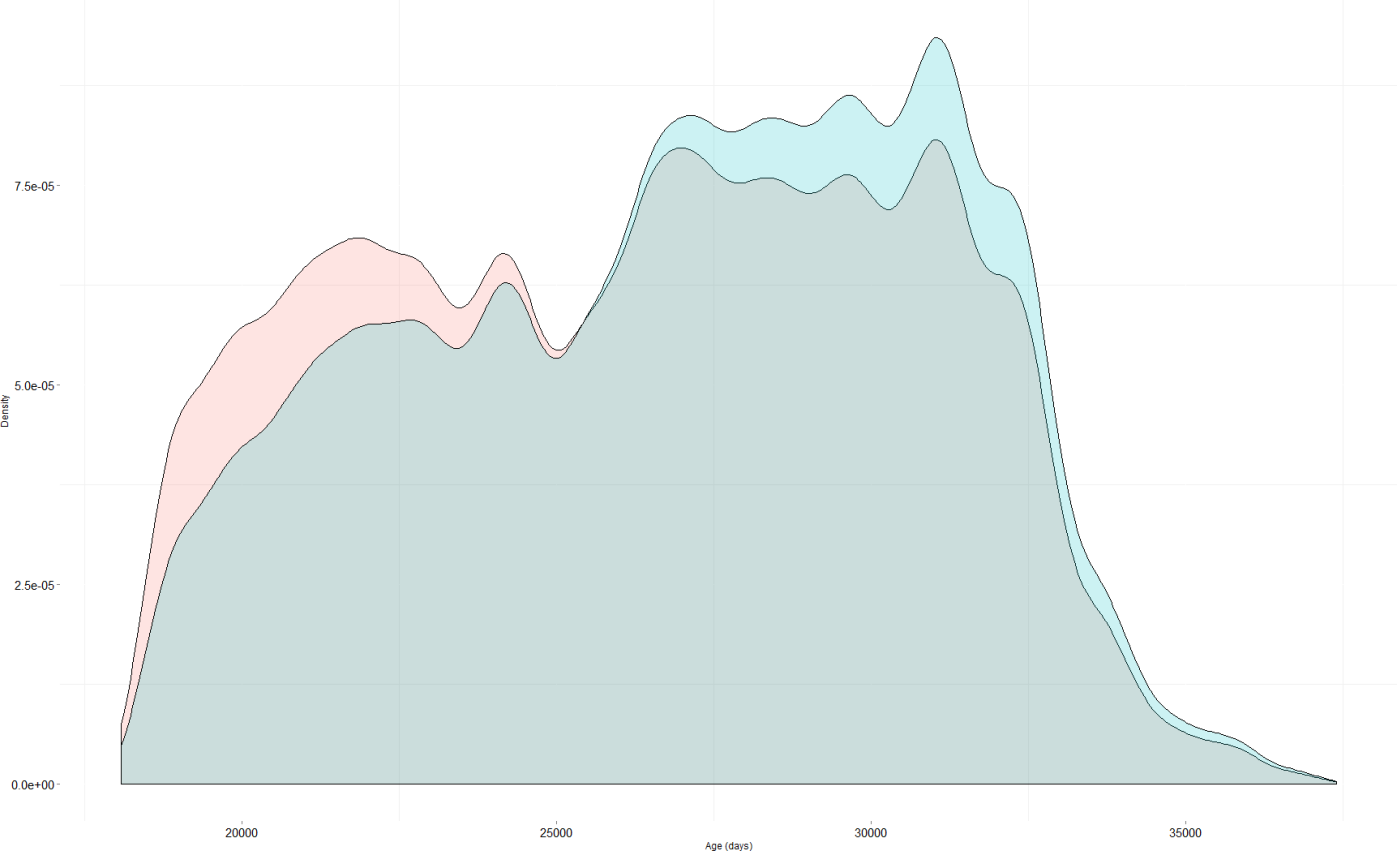
Density plot for age, in days, from random sampling (red, *M*_*t*_) and from covariate (age)-dependent sampling (blue, *M*_*th*.*age*_).

In the scenario of covariate-dependent sampling older cases were overrepresented in the final dataset as compared to random sampling. Older cases were also more likely to be detected multiple times in this scenario (Fig 1).

#### Effect of spatial heterogeneity (*M*_*th*.*spat*_, *M*_*th*.*age*.*spat*_, *M*_*thb*.*spat*.*ref*_, *M*_*thb*.*age*.*spat*.*ref*_)

**Fig 2:**
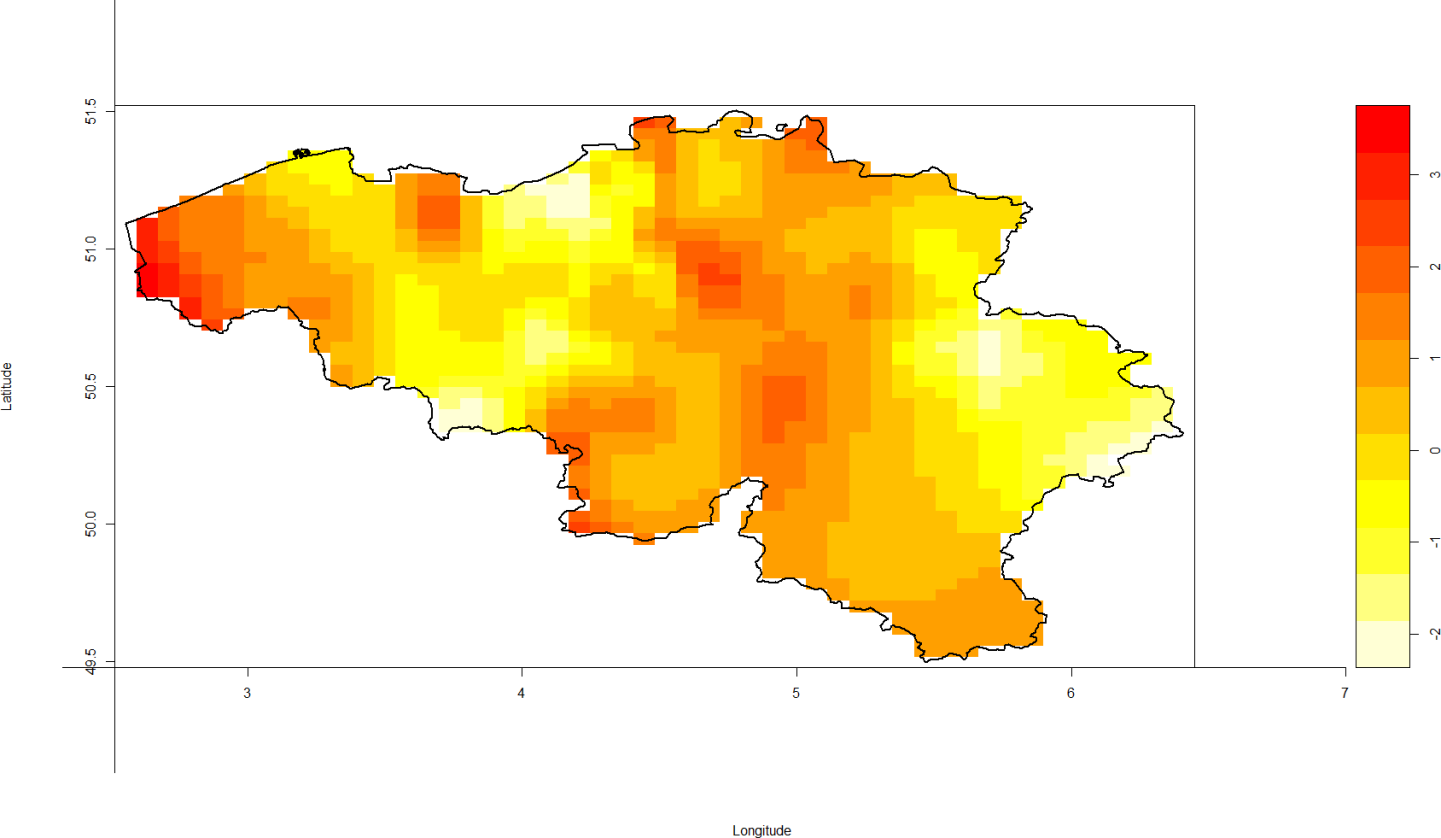
The heat plot represents the log of the ratio of the densities of spatially dependent and random sampling. Spatial heterogeneity was based on the relation between the location of the labs and the location of the generated cases. Yellow zones represent regions where cases are less likely detected by lab-surveillance (either NRC or sentinel surveillance) in the spatial heterogeneity scenario, as compared to random sampling.

The subset of labs and hospitals that participated in surveillance was not equally distributed and there was dependence between sentinel and NRC surveillance. Labs that participated in sentinel surveillance were also more likely to participate in NRC surveillance, as compared to labs that did not participate in sentinel surveillance (OR = 12.73, p<0.0001). One of the consequences of this spatial heterogeneity was uneven representation of regions (Fig 2).

#### Effect of referrals between sources (*M*_*tb*.*ref*_, *M*_*thb*.*age*.*ref*_, *M*_*thb*.*spat*.*ref*_, *M*_*thb*.*age*.*spat*.*ref*_)

Referrals between sources resulted in dependence between sentinel and NRC surveillance and hospital and NRC surveillance. Cases detected by sentinel surveillance were also more likely detected by NRC surveillance, as compared to cases not detected by sentinel surveillance (e.g. OR = 1.719, sd = 0.114, averaged over the simulation runs).

The scenario did not include referrals between the hospital study and the sentinel surveillance. However because of the referrals the “three way”-interaction was not zero. The “three way”-cross product of the cell counts λ; ($\frac{\lambda_{111}\;\lambda_{100}\;\lambda_{010}\;\lambda_{001}}{\lambda_{011}\;\lambda_{000}\;\lambda_{110}\;\lambda_{101}}=0.897$, sd = 0.149, averaged over the simulation runs, notation in S1 Appendix) was not one. This meant that the referrals have introduced dependence between sentinel surveillance and the hospital study for cases also observed in NRC surveillance.

### CR estimators without assumptions on the underlying dependency structure

An overview of the models, selected by AIC, can be found in Table 2.

**Fig 3:**
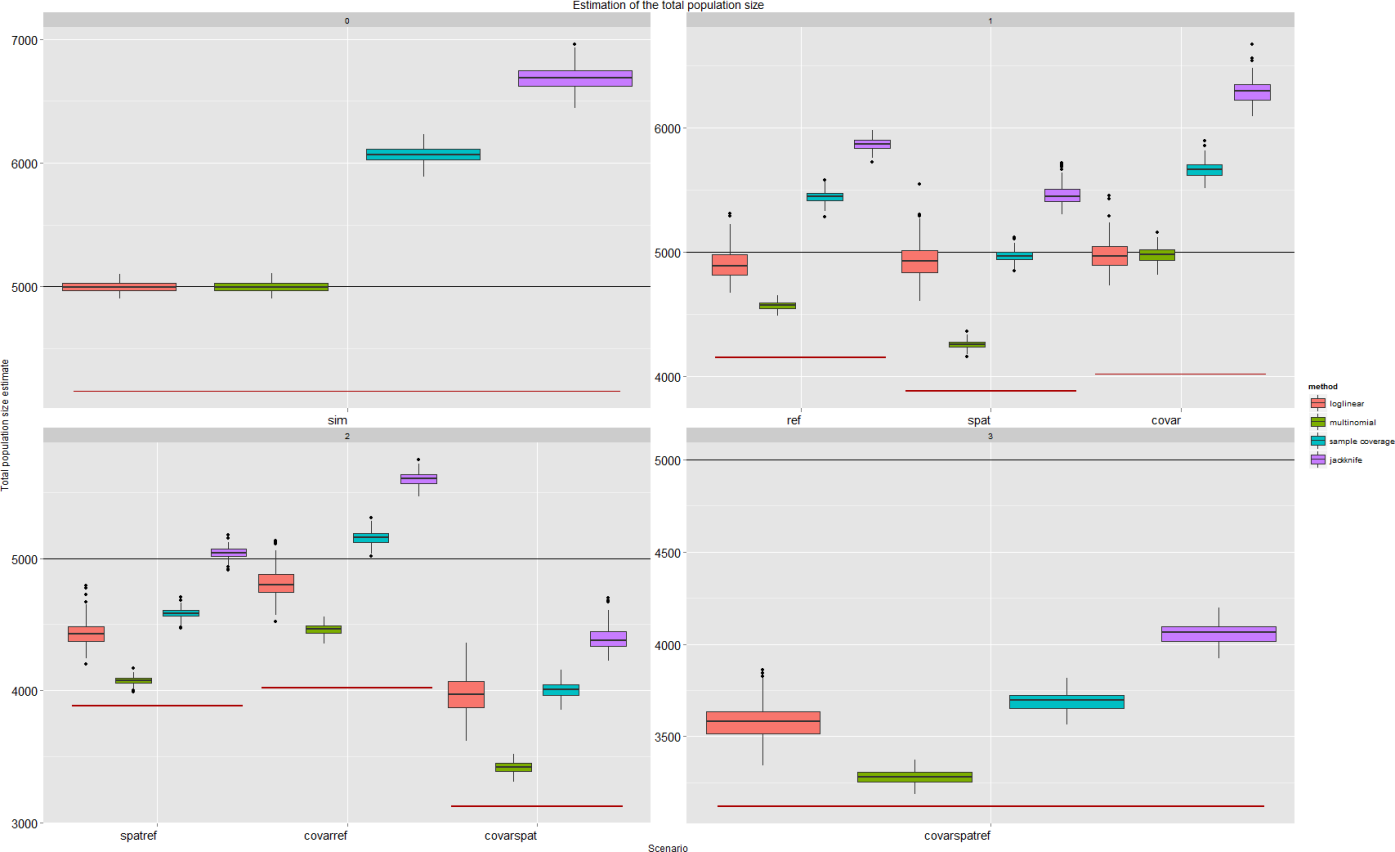
Boxplots of the obtained estimates per scenario and method. The models were chosen without assumptions on the underlying dependency structure. The black line indicates the total population size, the red line indicates the number of unique cases per scenario.

After the inclusion of the age covariate in the multinomial model there was no residual unmodelled heterogeneity in the *M*_*th*.*age*_-scenario. The odds ratio increase per unit (day) was 1.000125 (estimate = 0.12e-3, se = 3.173e-6, averaged over the simulated runs). Other methods could not fully correct for covariate-dependent sampling. Inclusion of the distance covariate did not completely correct for spatial heterogeneity. The parameter for distance was significant and did show the lower detection probability with increasing distance to the detecting lab or hospital. The odds ratio decrease per unit (meter) was 0.999 (estimate = -1.746e-5, se = 3.173e-6 averaged over the simulated runs). The log-linear model was the only model that fully corrected for the referrals. Multinomial likelihood modelling only performed better than log-linear modelling if covariates, with a strong influence on the individual detection probability, were part of the conditional likelihood model as was the case in the *M*_*th*.*age*_-scenario, but not in the *M*_*th*.*spat*_- and *M*_*tb*.*ref*_- scenarios. As more dependence was introduced, the estimates in the likelihood framework had a larger bias. In the most complex scenario (*M*_*thb*.*covar*.*spat*.*ref*_) log-linear modelling, including all two-way interactions, underestimated the total population size by, on average, 1416 cases (28%). Multinomial modelling, conditional on the distance and age covariates, underestimated the total size by 1740 cases (25%) in the same scenario. The non-parametric methods had the largest bias in scenarios without dependence. The relative bias of all the capture-recapture estimators was smaller than the relative bias of the unique cases-estimator in the scenarios with two and three sources of dependence (S1 Appendix). The jackknife-estimator had a larger relative bias than the unique cases-estimator in two of the three scenarios with one source of heterogeneity (*M*_*tb*.*ref*_ and *M*_*th*.*age*_) and in random sampling (*M*_*t*_). The sample coverage-estimator had a larger relative bias in the *M*_*t*_-scenario (Fig 3).

### CR estimators with assumptions on the underlying dependency structure

**Fig 4:**
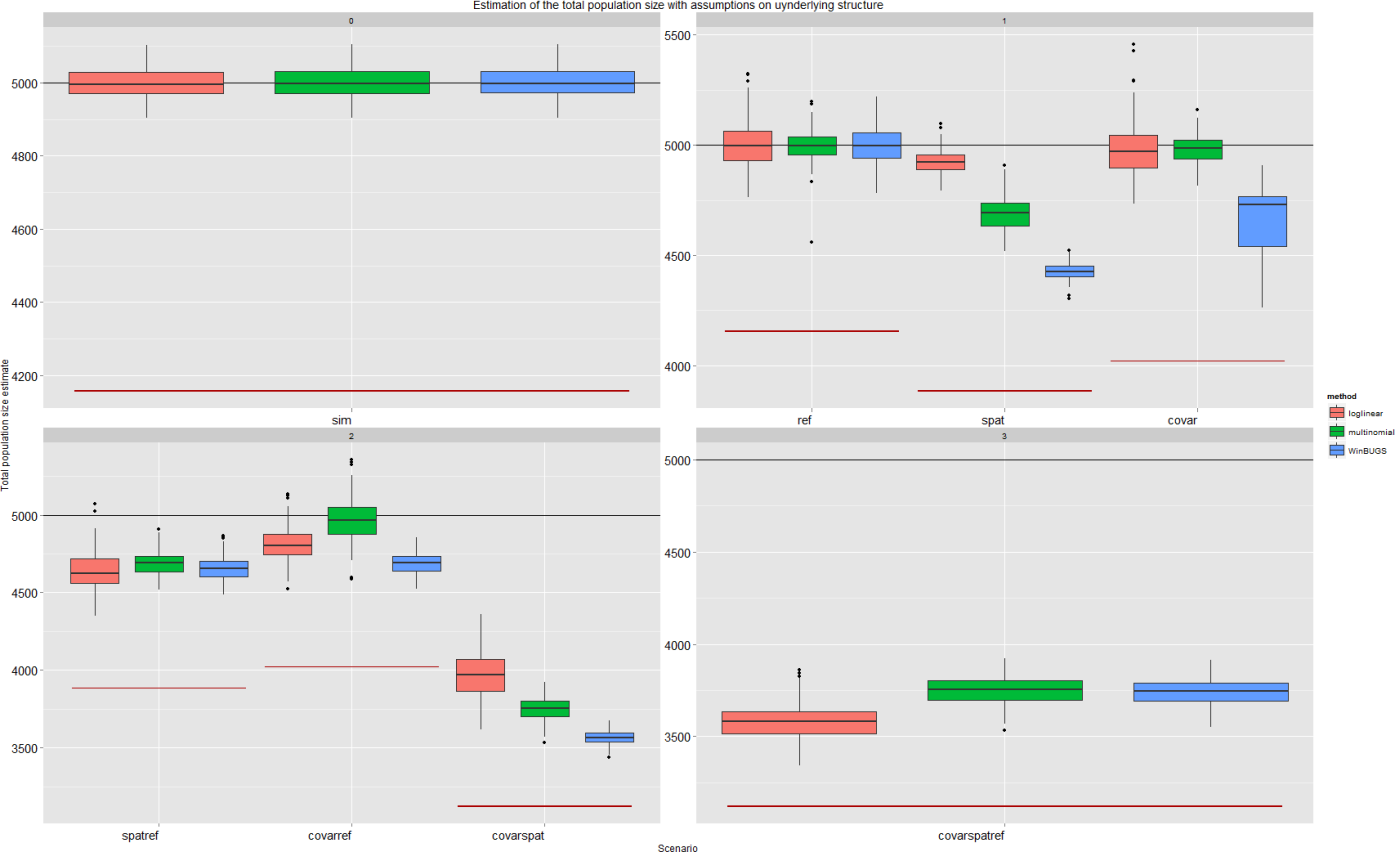
Boxplots of the obtained estimates per scenario and method. The models were chosen based on assumptions about the underlying dependency structure. The black line indicates the total population size, the red line indicates the average number of unique cases per scenario.

**Fig 5:**
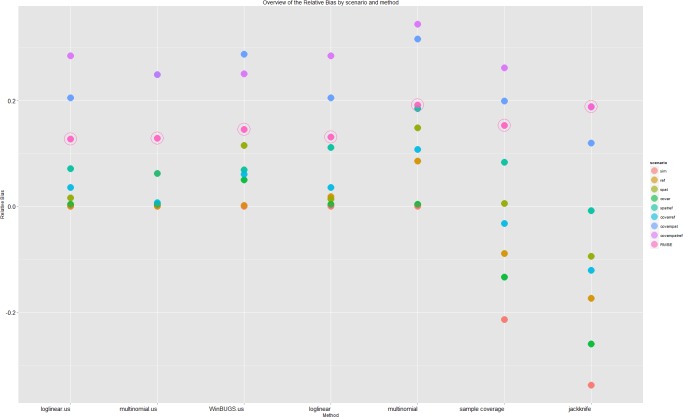
Overview of the relative bias by method and scenario. The dots represent the average relative bias over the simulation runs. The root mean squared average relative bias (RMSE) per method is also presented with an extra circle around the dot.

Parameter estimability limited the estimation of more than one “recapture” probability. In three scenarios we selected a log-linear model with a higher AIC (*M*_*thb*.*spat*.*ref*_, *M*_*th*.*spat*_, *M*_*tb*.*ref*_), only in the *M*_*thb*.*spat*.*ref*_-scenario did this result in a better estimate. During model selection we did not take non-hierarchical models into account (Fig 4). Allowing models to estimate a second detection probability conditional on previous detections resulted in better estimates for multinomial models (**Fig 5**).

Referrals were correctly estimated by the WinBUGS model at 30% and 40%, unless a recapture probability also needed to be estimated in which case these estimates were confounded. In our current WinBUGS model individual covariates could not be incorporated, this resulted in a larger, as compared to the log-linear and multinomial models, bias in covariate-dependent sampling.

An overview of selected models can be found in Table 3.

**Table 3:** Models with assumptions about the underlying dependency structure. For log-linear models a selection was made out of all hierarchical models. For the multinomial models a selection was made out of all possible models (including models with probabilities conditional on covariates, mixtures and previous detections). *p*_1_ = p(sentinel), *p*_2_ = p(hospital), *p*_3_ = p(NRC), distance in meters, age in days, q = referrals, r = (re)detection probability conditional on previous detection, *p*_3_*s*_11_ = probability of detection in sample 3 conditional on detection in sample 1, *p*_3_*s*_10_ = probability of detection in sample 3 conditional on absence in sample 1, c = covariate-effect (notation in S1 Appendix).

Scenario	Log-linear	Multinomial Likelihood	Bayesian approach
*M*_*t*_	∼ *p*_1_,*p*_2_,*p*_3_	∼ *p*_1_,*p*_2_,*p*_3_	∼ *p*_1_,*p*_2_,*p*_3_
*M*_*tb*.*ref*_	∼ *p*_1_,*p*_2_,*p*_3_, *p*_1_ * *p*_3_, *p*_2_ * *p*_3_	∼ *p*_1_,*p*_2_,*p*_3_,*r*_3_	∼ *p*_1_,*p*_2_,*p*_3_,*q*_13_,*q*_23_
*M*_*th*.*spat*_	∼ *p*_1_,*p*_2_,*p*_3_, *p*_1_ * *p*_3_	*M*_*t*_ ∼ *p*_1_,*p*_2_,*p*_3_,*r*_3_, *distance*	∼ *p*_1_,*p*_2_,*p*_3_*s*_11_,*p*_3_*s*_10_
*M*_*th*.*age*_	∼ *p*_1_,*p*_2_,*p*_3_, *p*_1_ * *p*_3_, *p*_2_ * *p*_3_, *p*_1_ * *p*_2_	*M*_*t*_ ∼ *p*_1_,*p*_2_,*p*_3_,*r*_3_, *age*	∼ *p*_1_,*p*_2_,*p*_3,_,*c*
*M*_*thb*.*spat*.*ref*_	∼ *p*_1_,*p*_2_,*p*_3_, *p*_1_ * *p*_3_, *p*_2_ * *p*_3_	*M*_*t*_ ∼ *p*_1_,*p*_2_,*p*_3_,*r*_3_, *distance*	∼ *p*_1_,*p*_2_,*p*_3_*s*_11_,*p*_3_*s*_10_,*q*_13_,*q*_23_
*M*_*thb*.*age*.*ref*_	∼ *p*_1_,*p*_2_,*p*_3_, *p*_1_ * *p*_3_, *p*_2_ * *p*_3_, *p*_1_ * *p*_2_	*M*_*t*_ ∼ *p*_1_,*p*_2_,*p*_3_,*r*_3_, *age*	∼ *p*_1_,*p*_2_,*p*_3_,*q*_13_,*q*_23_,*c*
*M*_*th*.*age*.*spat*_	∼ *p*_1_,*p*_2_,*p*_3_, *p*_1_ * *p*_3_, *p*_2_ * *p*_3_, *p*_1_ * *p*_2_	*M*_*t*_ ∼ *p*_1_,*p*_2_,*p*_3_,*r*_3_, *age*	∼ *p*_1_,*p*_2_,*p*_3_*s*_11_,*p*_3_*s*_10_
*M*_*thb*.*age*.*spat*.*ref*_	∼ *p*_1_,*p*_2_,*p*_3_, *p*_1_ * *p*_3_, *p*_2_ * *p*_3_, *p*_1_ * *p*_2_	*M*_*t*_ ∼ *p*_1_,*p*_2_,*p*_3_,*r*_3_, *age*	∼ *p*_1_,*p*_2_,*p*_3_*s*_11_,*p*_3_*s*_10_,*q*_13_,*q*_23_

### CR estimators under increasing, but random, data reduction

**Fig 6:**
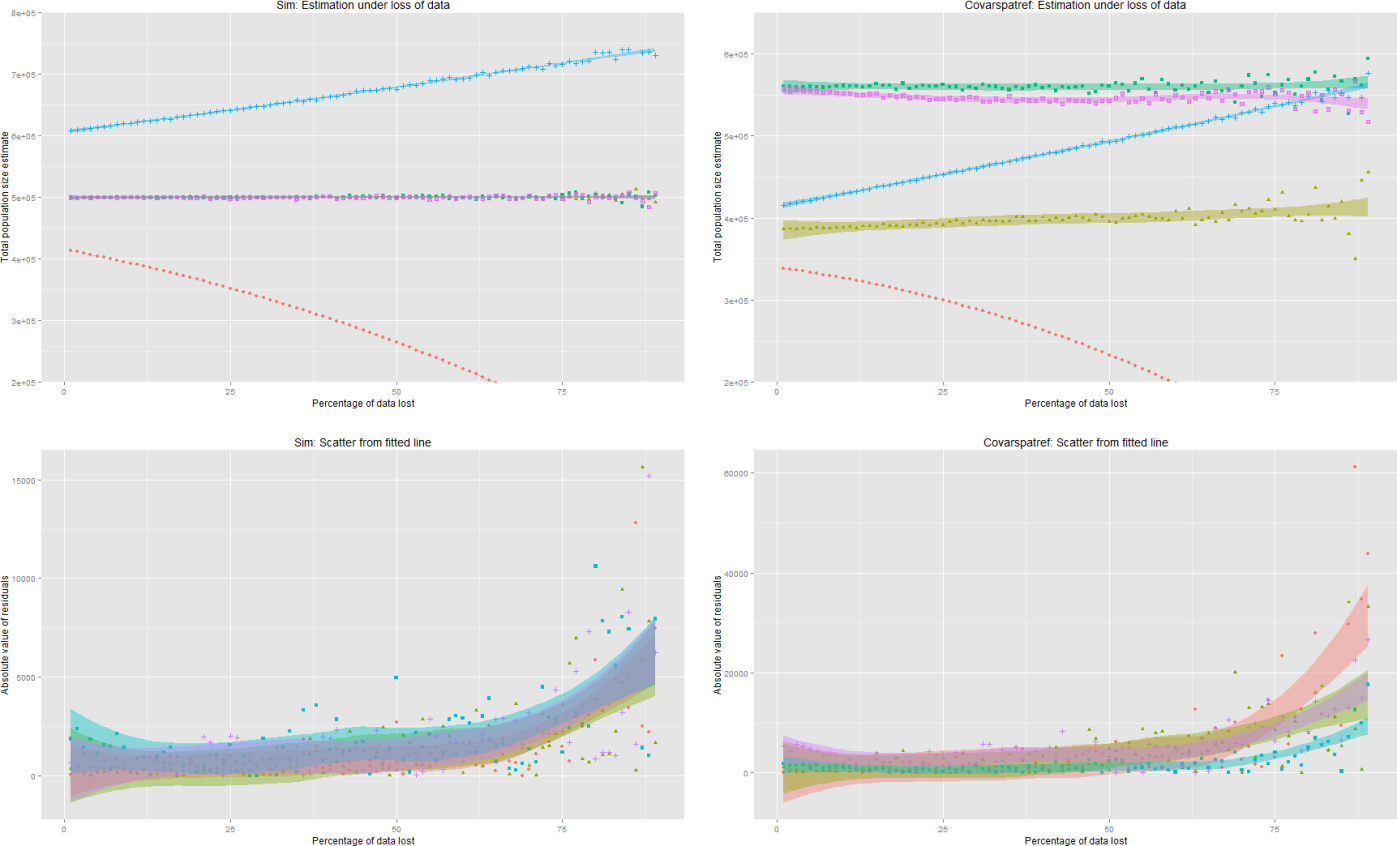
Overview of total population size estimation and absolute residuals by method (“loglinear” = red, “multinomial” = green, “bayesian method” = purple, “sample coverage” = cyan, “number of detected cases” = red) under random, increasing loss of data.

The trend of the total population size estimates under a random, but increasing, loss of data was significantly increasing in both scenarios for Chao’s sample coverage (estimate = 118.75, p<0.001, *M*_*thb*.*covar*.*spat*.*ref*_). In the *M*_*thb*.*covar*.*spat*.*ref*_-scenario the log-linear estimator also showed a significantly increasing trend (estimate = 72.41, p<0.001, *M*_*thb*.*covar*.*spat*.*ref*_). As data was reduced the estimation becomes unstable with increasing scatter around the fitted line. This became more apparent as 50% or more of the data per source was lost (**Fig 6**).

## Results of the estimation of the number of IPD and pertussis cases

### Description of the IPD and Pertussis datasets

#### IPD

**Fig 7:**
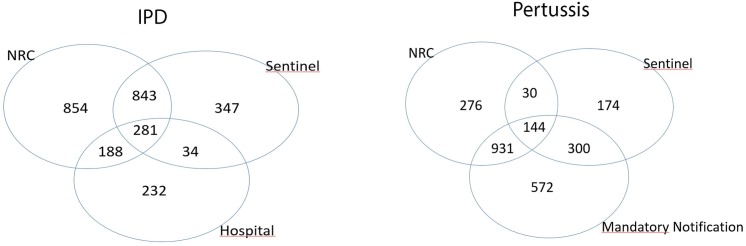
Overview of the IPD (left) and pertussis (right) data after matching.

A total of 1505 IPD cases were reported through sentinel surveillance, 2166 through the NRC and 735 through the hospital study. The number of unique cases after matching was 2779 (**Fig 7**, left).

#### Pertussis

A total of 648 pertussis cases were reported through sentinel surveillance, 1381 through the NRC and 1947 through mandatory notification. The number of unique cases after matching was 2427 (**Fig 7**, right).

### Estimation of the total population size

#### IPD

**Fig 8:**
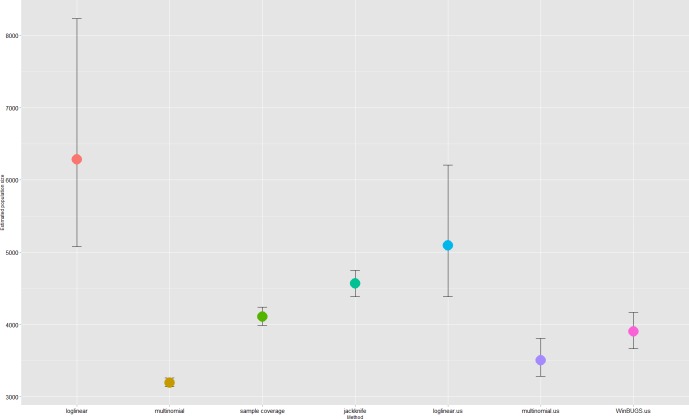
Overview of the seven different estimators for the IPD study and their 95% confidence intervals.

Experts mentioned the following possible components of the underlying dependency structure. The laboratories of the hospitals participating in the hospital study were encouraged to send isolates to the NRC for conformation and further typing. The same recommendation was made to the sentinel labs. There was interaction between the sentinel surveillance and the NRC because their detectors overlap (see “Effect of spatial heterogeneity”). These three components were modelled in the multinomial likelihood by defining a separate recapture probability for the NRC conditional on a previous detection in the hospital and/or sentinel surveillance. The multinomial model without recapture probability was also fitted. In log-linear modelling two interaction terms were included: sentinel surveillance*NRC, hospital study*NRC. A log-linear model with all three two-way interactions was also presented as this model had the lowest AIC. The WinBUGS-model allowed for the direct modelling of the two above suggested referrals and the interaction (see S1 Appendix). The estimated total population varied between 3512 (Multinomial with recapture probability for NRC) and 4580 (Burnham’s jackknife) for the four estimators that showed the best performance in the most complex scenario in the simulation studies (also including Chao’s sample coverage (N = 4109) and the WinBUGS-model (N = 3905)) (**Fig 8**).

The effect of age on conditional detection probabilities in the multinomial model was not significant if it was fitted as a common parameter. If separate parameters were estimated by detection probability, a small but significant negative effect on the conditional detection probability in the hospital study was found. The probability to be included in the hospital study was 11% for a case aged 70 years and 12% for a case aged 80 years (log odds ratio estimate = -8.9e-03 CI(-1.6e-02, -1.9e-03)). The covariate distance shows a significant effect on detection probability in all three lists (log odds ratio estimate = -6.96e-06 CI(-1.07e-05–3.19e-06)). When the probabilities were modelled separately, the recapture probability (for detection by NRC) was not distance dependent (log odds ratio estimate = -1.63e-06 CI(-7.09e-06–3.83e-06)). The referrals were estimated by the WinBUGS-model at 0.46 for the sentinel and NRC surveillance and at 0.14 for the hospital study and NRC surveillance.

#### Pertussis

Experts mentioned the following possible components of the underlying dependency structure. Both the NRC and the sentinel surveillance were expected to notify cases to the mandatory notification system. This dependence was modelled with a multinomial model by defining a separate recapture probability for the mandatory notification conditional on a previous detection in the NRC and/or sentinel surveillance. As in the IPD study, there was interaction between the sentinel surveillance and the NRC because their detectors overlap. The variance components of a model that also defined a separate recapture probability for the NRC conditional on being detected in the sentinel sample, could not be estimated. The multinomial model without recapture probability was also fitted. In loglinear modelling two interaction terms were included: sentinel surveillance*NRC, hospital study*NRC. The model with all three two-way interactions was also presented, as it had the lowest AIC. The WinBUGS-model allowed for the direct modelling of the two above suggested referrals and the interaction between sentinel and NRC surveillance.

**Fig 9:**
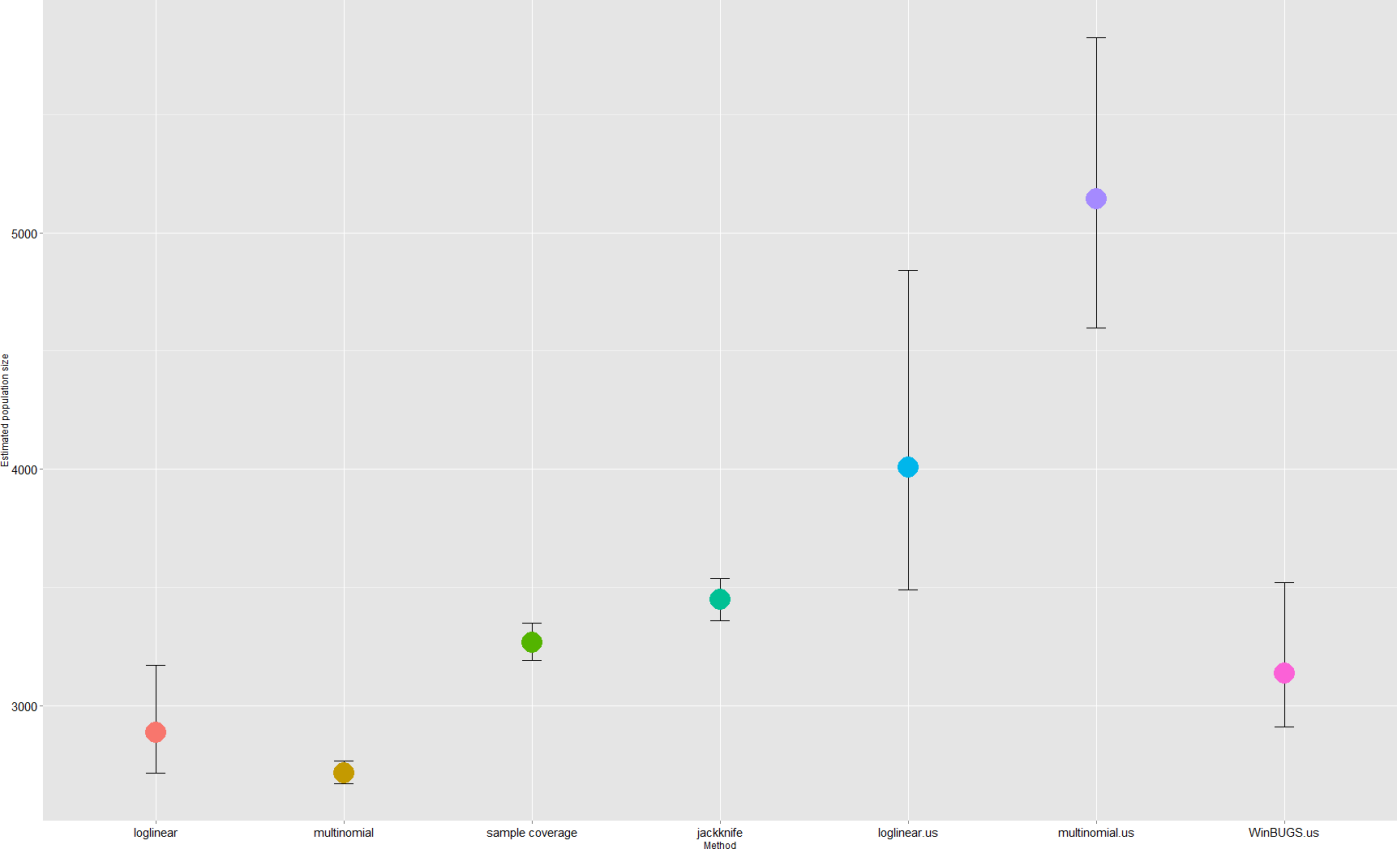
Overview of the seven different estimators for the pertussis study and their 95% confidence intervals.

The estimated total number of pertussis cases varied between 2714 (Multinomial *M*_*t*_ model with recapture probability for mandatory notification) and 3449 (Burnham’s jackknife) for the four estimators that performed best in the simulation study (also including Chao’s sample coverage (N = 3267) and the Bayesian approach (N = 3138)) (**Fig 9**).

Age did not have a significant influence on the detection probability of pertussis cases as the parameter did not reach significance in a multinomial logit model. The referrals were estimated at 16% for the sentinel surveillance to the mandatory notification and at 56% for the NRC surveillance to the mandatory notification.

## Discussion

### Strengths

This was not the first study to evaluate the performance of capture-recapture estimators using a simulation study [35–37]. To our knowledge, however, we were the first to investigate the performance of different estimators under a systematically increasing violation of assumptions. This was also the first study in which dependencies between datasets were based on aspects of actual surveillance networks for infectious diseases (such as the spatial configuration of labs participating in sentinel surveillance and referrals between networks). We also focused on testing the suggestion that assumptions about the likely relationships between data sources should be central in model building and selection. This has been an important suggestion made by previous studies [6,9,10]. Apart from the bias of the different estimators in increasingly complex scenarios, we thus also evaluated the opportunities these estimators offered for modelling dependencies and the bias of models selected by assumptions. Finally, the applicability of the estimators was evaluated with two three-sample datasets; one on IPD and one on pertussis.

### Lessons learned

Our simulation study indicated that while no capture-recapture estimator existed that remained unbiased in all scenarios, almost all had a lower bias than an estimation of the total population by the sum of unique cases. The WinBUGS-model, the multinomial model with “recapture”-probabilities, Burnham’s jackknife and Chao’s sample coverage estimated the total population size within 75–82% of the actual size in the most complex scenario. The non-parametric estimators did not perform well in the less complex scenarios. They only performed well if there was a certain degree of dependence between sources. Parametric estimators were relatively robust to random data loss, but variation in the residuals increased as more than 50% of the data was lost. Chao’s sample coverage showed a linear increasing trend over random data loss.

### The IPD and pertussis datasets

We selected the four methods that performed best in the simulation study to calculate incidence estimates. For adults aged 50 years and older that were diagnosed with IPD in 2010 in Belgium the lowest and highest estimates were 44/100,000 and 58/100,000 respectively. The lowest incidence estimate for Belgian pertussis cases in 2014 was 24.2/100,000 and the highest 30.8/100,000. The proportion of undetected cases was larger for IPD (26–65% vs. 12–42% for pertussis). This is an indication that surveillance for pertussis was more exhaustive.

In every capture-recapture study the plausibility of the estimates should be critically evaluated. One way to do this is by comparing estimates to internationally reported rates and incidences [38–41]. The European average reported rate was 15.6/100,000 in 2010 for adults aged 65 years and older [42,43]. These are however reported rates. They are unadjusted for undetected cases. Several other items or practices might differ between countries and influence incidence estimation. These include, but are not limited to, microbiological diagnostic techniques, vaccination coverage and case definition.

Capture-recapture studies are not limited to total population size estimation. They can also offer insight into the interactions between surveillance networks and the role of case covariates. One of our additional findings was a lower capture probability for older patients in the IPD-hospital sample. This may indicate that older IPD cases were not always hospitalised. This finding is in accordance with statements by experts. We also observed that distance was of importance in the IPD study as recapture probability decreased with increasing distance. The effect of distance was however smaller than in the simulation study. Possible explanations for this were tertiary centres and disease severity. A case with a more severe IPD will be detected easier while also having the tendency to go further (to a tertiary centre). Secondly, anyone going to a tertiary centre, which is often not the closest hospital, is likely to be detected since tertiary centres are good reporters. The referrals estimated by the WinBUGS-model were much lower than the degree of referrals suggested by the experts. The referrals from the hospital study to the NRC surveillance were estimated at 16%. Even though this parameter was confounded with the capture and recapture probabilities, such findings could lead to further inspection.

### A parametric or non-parametric framework

In the absence of knowledge or assumptions on the underlying dependency structure, non-parametric estimators outperform likelihood based estimators in the more complex scenarios. Previous (simulation) studies have come to a comparable conclusion [14,44]. Chao and Lee have formulated a guideline for the selection of a parametric or non-parametric framework [45].

#### Parametric methods: Log-linear models and the issue of model selection/building

A set of independent Poisson random variables gives a multinomial distribution when conditioned on their sum. Log-linear Poisson models are thus equivalent to conditional multinomial likelihood models [46]. Log-linear and multinomial models do however offer different modelling possibilities. A multinomial logit model allows for continuous covariates and a limited number of conditional recaptures. While this proved to be helpful in a scenario with covariate-dependent sampling or referrals, the bias was large in the scenario with spatial heterogeneity. The number of additional conditional recaptures necessary to obtain a better estimate in this scenario was too high, leaving such models parameter redundant [47]. A log-linear model allows for interaction between detection probabilities, but continuous covariates cannot be used and an all interactions, full rank model is not identifiable. Categorical covariates can be included to reduce bias, but it might be unclear how to best categorise continuous covariates [17]. Dependencies are confounded in the interaction term and there is thus no need to state them explicitly. Since an ‘all interactions’-model is parameter redundant, researchers often set the all-source interaction to zero. We showed that a violation of this assumption, as in scenarios with multiple sources of dependence, led to a biased estimator. If non-standard, non-hierarchical models are considered the all-source interaction term can be estimated instead of one of the lower order-interaction terms. One of the problems accompanying these models is that several plausible models might have an equally good fit, but different estimates. As a possible alternative, Jones et al. suggested to combine the multinomial and log-linear approaches by explicitly modelling the dependencies. While this approach has its advantages, explicit assumptions are now more important, rendering the models less robust. They chose the program WinBUGS for pragmatic reasons [6]. Future studies should try to extent their approach and include hierarchical and logit models.

In our simulation study, AIC as information criterion for model selection resulted in the selection of saturated, sometimes overfitted models. If there is unmodelled dependence between sources extra binomial variation occurs and AIC, unadjusted for overdispersion, has the tendency to select overfitted models [48]. This is problematic since previous studies have found all “two way” interaction log-linear models to estimate abundance too high [5]. Overall, standard model selection methods (such as AIC) are not completely suitable for capture-recapture studies since these methods assess the fit of the model to the observed data, while the interest lies in the unobserved data.

### Weaknesses of this study

#### The simulation study

Our study is subject to several limitations. The perhaps most apparent is the impossibility to translate the simulation study to actual datasets. Though the scenarios are based on components of the underlying dependency structure of datasets, the simulation study only investigates the performance of estimators under its own specific characteristics. This is a known issue in all simulation studies [44].

The underlying dependency structure of a real dataset might have additional components. We did not consider dependence between cases. For an infectious disease it can be expected that a patient infects those closed to him. It can also be expected that those persons will go to the same hospitals and samples will be send to the same laboratory [49]. We did also not consider heterogeneous capture probabilities conditional on unrecorded covariates or by a certain time trend. Additionally selective forwarding of the data might be possible.

#### The datasets: Quality of the data/case definition

Despite an active pursuit for complete case information, some of the case records were incomplete. If necessary variables for the matching of cases were missing, cases were discarded. Some patients did not give informed consent for the use of their data in the IPD hospital study. The investigators leading the IPD hospital study suggested that this was more often so for older patients. This is an important remark with respect to the finding that older cases were less likely captured in the hospital study.

Epidemiologist performing capture-recapture studies should be aware of the consequences of case definition, e.g. the IPD case definition allows only for the incidence estimation of laboratory-confirmed IPD cases. Though laboratory confirmed-cases may be a good proxy for IPD-cases, it may be hard to estimate the burden of disease for other infectious diseases with a similar method. Cases with a non-existent capture probability cannot be estimated. We did not take possible misidentification into account; 100% sensitivity and 100% specificity were assumed for the laboratory diagnosis.

#### The datasets: Matching

We did not adjust for possible incorrect matching. The assumption of perfect matching is strong, certainly if readily available epidemiological datasets are used, which are often (out of ethical considerations) made anonymous. A small sensitivity analysis does however indicate that it was justifiable (S1 Appenidix). Additionally, some cases were still recorded manually and the period between detection dates from which we decided whether identical cases were matches was determined arbitrarily.

#### Limited number of estimators

This study only considered closed population methods for three sample-studies. We limited the methods to five methods. We selected these specific methods because they had already been applied to epidemiological datasets and for their convenience. Spatially-explicit capture-recapture (SECR) models were not included in this study. SERC-models combine the probability of capture with the probability of presence [50,51]. Their use in epidemiology needs to be explored. A drawback of SECR models is that they are computationally intensive. Another method that may prove performant in scenarios with spatial heterogeneity is mark-recapture distance sampling, the current software however only allows for two samples [52]. To investigate the influence of covariates on capture probabilities, we limited the modelling of the conditional detection probabilities to logistic regression. Other, more flexible, methods were available [53]. Because we wanted to limit the estimators to readily available estimators we did not extent the approach of Jones et al. to multinomial logit models.

Finite mixture models were allowed into the multinomial likelihood methods used in this study, but did not result in better estimates. We did not thoroughly investigate this. Problems with the identifiability of the population size with mixing models are a well-known issue. Multimodality or a flat likelihood can make the choice of the mixture proportions quite random. Link et al. examined the identifiability problem with mixture models; especially when the probability of capture is small or the degree of heterogeneity is large, the log-likelihood surface is relatively flat and it is difficult to obtain much information about the total population size [4,54,55]. The available methodology is not limited to finite mixture models [56]. Pledger and Phillpot offer a comprehensive review of the use of random effects and mixture models [57].

#### Parameter estimability

Experts suggested more complex models for the IPD and pertussis study. Models including for example an additional referral parameter, the NRC refers some of its cases to the sentinel network, were parameter redundant. The limited number of estimable parameters is an important consideration in capture-recapture studies. This is an issue in multinomial and log-linear models alike. In both it will, for example, limit the number of categories of a categorical covariate.

## Conclusion

The performance of log-linear, multinomial and non-parametric capture-recapture estimators is sensitive to the assumption of homogeneous capture probabilities. Dependence between samples does however not exclude unbiased estimation. Parametric methods offer modelling opportunities to (partly) compensate for heterogeneity. Non-parametric methods inherently compensate for part of this heterogeneity. Parametric methods are flexible, but limited by parameter estimability and the necessity of additional knowledge such as individual covariates and assumptions about the underlying dependency structure. Except for non-parametric estimators in the simplest scenarios, the estimators had a lower relative bias than the estimate obtained by summing up the unique cases.

We encourage the use of capture-recapture methods, but epidemiologists should preferably include datasets for which the underlying dependency structure is not too complex, a priori investigate this structure, compensate for it within the model and interpret the results with the remaining unmodelled heterogeneity in mind.

## Supporting Information

S1 Appendix (DOCX)
