# Correction: Capture-Recapture Estimators in Epidemiology with Applications to Pertussis and Pneumococcal Invasive Disease Surveillance

**DOI:** 10.1371/journal.pone.0165351

**Published:** 2016-10-18

**Authors:** Toon Braeye, Jan Verhaegen, Annick Mignon, Wim Flipse, Denis Pierard, Kris Huygen, Carole Schirvel, Niel Hens

The second author’s name is spelled incorrectly. The correct name is Jan Verhaegen. The correct citation is: Braeye T, Verhaegen J, Mignon A, Flipse W, Pierard D, Huygen K, et al. (2016) Capture-Recapture Estimators in Epidemiology with Applications to Pertussis and Pneumococcal Invasive Disease Surveillance. PLoS ONE 11(8): e0159832. doi:10.1371/journal.pone.0159832.
