# Supplementary material for: Capture-Recapture Estimators in Epidemiology with Applications to Pertussis and Pneumococcal Invasive Disease Surveillance
Source: PLoS One. 2016 Aug 16;11(8):e0159832. doi: 10.1371/journal.pone.0159832 (PMC4987016; doi:10.1371/journal.pone.0159832)
Supplement: S1 Appendix — (DOCX) [file pone.0159832.s001.docx]

# Supporting information file

Contents

[Supporting information file 1](#_Toc458061844)

[1. Terminology & Notation 1](#_Toc458061845)

[2. Sensitivity of the matching algorithm 2](#_Toc458061846)

[Simulated datasets: 2](#_Toc458061847)

[Influence of the interval between detection dates: 2](#_Toc458061848)

[Influence of typos: 3](#_Toc458061849)

[3. A short description of the used capture-recapture estimators 3](#_Toc458061850)

[Log-linear and multinomial models (Likelihood framework) 3](#_Toc458061851)

[Direct modelling approach suggested by Jones et al. (Bayesian framework) 4](#_Toc458061852)

[Chao’s sample coverage, Burnham’s jackknife (Non-parametric framework) 4](#_Toc458061853)

[4. Relative bias, standard error and RMSE 5](#_Toc458061854)

[5. WinBUGS-code 8](#_Toc458061855)

[6. References 10](#_Toc458061856)

## Terminology & Notation

**Captured:** The registration of a case in a certain sample is a “capture”, conform to terminology used in capture-recapture studies for animal abundance.

**Capture history:** The presence of a case in the separate samples is recorded by its capture history.

**Heterogeneity and homogeneity:** If sampling is random, the cases have a homogeneous capture probability. If not all cases are equally probable to be present in a sample, capture probability is heterogeneous.

**Underlying dependency structure:** The effect that the relation between samples (referrals and interactions) and heterogeneous capture probabilities have on the final dataset of unique cases (obtained by merging the samples).

**Detectors:** All institutes who are able to detect cases are called detectors, in our study these are laboratories and hospitals. Not all detectors of cases are submitting cases to one of the samples, those that do are detectors participating in a surveillance system.

**Local dependence:** Samples that are not independent because of interactions between samples and/or referrals between samples.

**Referrals:** Cases are automatically detected by the sample they are referred to. Referrals result in local dependence.

**Notation:** Based on the work of Otis et al. [1], models are subdivided by the source of heterogeneity.

$M_{t}$= Capture probability per sample is different: $p_{1}\neq p_{2}\neq p_{3}$

$M_{h}$= Capture probability within a sample is different between cases: $p_{1}|c_{1}\neq p_{1}|c_{2}$ for $c_{1}\neq c_{2}$

$M_{b}$ = Capture probability is conditional on previous captures: $p_{3}s_{11}\neq$ : $p_{3}s_{10}$

$p_{1}$represents the probability of detection by sample 1. $c_{1}$represents a covariate associated with case 1. $p_{3}s_{11}$represents the detection probability of sample 3 conditional on being detected by sample 1. $p_{3}s_{10}$represents the detection probability of sample 3 conditional on not being detected by sample 1. The probability of detection conditional on a previous detection (without specifying in which sample) is $r$. $r_{3}=$probability of detection in sample 3 conditional on capture in sample 1 and/or sample 2.

**Contingency table:** The capture histories are often presented in contingency tables. We provide an example for a 2-sample study;

|  | Undetected in sample 1 | Detected in sample 1 |  |
| --- | --- | --- | --- |
| Undetected in sample 2 | $\lambda_{00}$ | $\lambda_{10}$ |  |
| Detected in sample 2 | $\lambda_{01}$ | $\lambda_{11}$(=$n_{12}$) | $n_{2}$ |
|  |  | $n_{1}$ |  |

## Sensitivity of the matching algorithm

The matching algorithm matched by the variables; “birthdate”, “gender”, “postcode”. An additional criterion was the interval between detection date in the different databases. We decided on 90 days as the interval over which cases with a matching “birthdate”, “gender”, “postcode” were regarded as different cases. After matching cases were merged the oldest detection data was kept in the database. As a consequence three matching cases were kept as two different cases if the detection date interval between the first and last case was larger than 90 days, even if the middle case could “bridge” the interval. (E.g. 40 day interval between case1 and case2 and 60 day interval between case2 and case3. Case1 and case2 were merged, case3 was kept as a separate case.)

To investigate the sensitivity of the matching procedure we undertook two short studies;

### Simulated datasets:

The algorithm was used on three of the simulated datasets. The algorithm did not result in false positives.

### Influence of the interval between detection dates:

For the NRC and hospital IPD-datasets 85% of matching cases were merged (using a 90 day interval). A 60 day-interval would result in 78% of the matching cases being merged. A 30 day-interval in 72%.

### Influence of typos:

Allowing one “Levenshtein distance” between the “birthdate, gender, postcode”-match key, in combination with the 90 day-interval between detection dates, resulted in two additional merges between the sentinel and hospital IPD-datasets (317 instead of 315).

## A short description of the used capture-recapture estimators

### Log-linear and multinomial models (Likelihood framework)

Darroch founded the mathematical framework for maximum likelihood estimation in closed populations in 1958 and extended it to open populations in 1959 [2]. Darroch represented the probability density of a certain capture history by a multinomial distribution.

Let $N$ be the unknown abundance (for other notation see appendix 1);

$$P(n_{12},n_{1}{,n}_{2}|N,p_{1},p_{2})=\frac{N!}{n_{12}!n_{1s20}!n_{2s10}!n_{0}!}{{(p}_{12})}^{n_{12}}{{(p}_{1s20})}^{n_{1s20}}{{(p}_{2s10})}^{n_{2s10}}{{(p}_{0})}^{n_{0}}$$

Under the assumption of independence of sources; (e.g.$p_{12}= p_{1}p_{2}, p_{1s20}= {p_{1}-p}_{12}=p_{1}{(1-p}_{2})$) the previously used “capture history”-probabilities can be reformulated to probabilities for being captured in the first sample or the second sample ($p_{1}{, p}_{2}$).

$$P\left( n_{12},n_{1}{,n}_{2} | N,p_{1},p_{2} \right)=\frac{N!}{n_{12}!{(n}_{1}-n_{12})!{(n}_{2}-n_{12})!(N-n)!}{{(p}_{1}p_{2})}^{n_{12}}{{(p}_{1}{(1-p}_{2}))}^{{(n}_{1}-n_{12})}{{(p}_{2}{(1-p}_{1}))}^{{(n}_{2}-n_{12})}{{((1-p}_{1}){(1-p}_{2}))}^{(N-n)}$$

There are three unknown parameters in the model; $N$,$p_{1}$ and $p_{2}$. The obtained Maximum Likelihood-estimators are the same as the Lincoln-Petersen estimates $p_{1}=\frac{n_{1}}{N}$ (estimated by $\hat{p}_{1}=\frac{n_{12}}{n_{2}}$) and $p_{2}=\frac{n_{2}}{N}$ (estimated by $\hat{p}_{2}=\frac{n_{12}}{n_{1}}$). Without the assumption of independence, separate conditional probabilities need to be estimated (e.g. $p_{1s20} and p_{1s21}$ instead of only $p_{1}$). In a two-sample study there are insufficient degrees of freedom to do this, hence the necessity to assume independence or constrain the parameters in some other way. The maximum likelihood estimator for $p$only has a closed-form solution in a two-sample study.

In the conditional multinomial likelihood approach, the unknown parameters$N, n_{0}$and $p_{0}$, present in the full multinomial likelihood, are conditioned out. The primary advantage of the conditional model is that individual covariates can be used to model capture probability. In a full likelihood model, covariates of unobserved animals would be necessary to model the probability of not being observed. This is not possible, covariates are only known conditional on being caught.

For a “two sample”-study, the probability that a person is recorded at least once is;

$$p_{.}=1-(1-p_{1})(1-p_{2})$$

The full likelihood can be formulated as follows;

$$P\left( n_{12},n_{1}{,n}_{2} | N,p_{1},p_{2} \right)=\frac{n!}{n_{12}!{(n}_{1}-n_{12})!{(n}_{2}-n_{12})!}\left( \frac{p_{1}p_{2}}{p_{.}} \right)^{n_{12}}({\frac{p_{1}{(1-p}_{2})}{p_{.}})}^{{(n}_{1}-n_{12})}({\frac{p_{2}{(1-p}_{1})}{p_{.}})}^{{(n}_{2}-n_{12})}$$

$$\frac{N!}{n!\left( N-n \right)!}\left( p_{.} \right)^{n}({1-p_{.})}^{\left( N-n \right)}$$

The first part is the conditional multinomial distribution. $\frac{N!}{n!\left( N-n \right)!}\left( p_{.} \right)^{n}({1-p_{.})}^{\left( N-n \right)}$ represents the binomial distribution for whether or not an animal was captured. The conditional maximum likelihood estimator N is asymptotically equal to the maximum likelihood estimator [3]. Once the capture probabilities are estimated, by maximizing the conditional likelihood, a “Horvitz-Thompson”-type estimator is used to calculated the population size [4].

$$\hat{N}=\frac{n}{\hat{p}_{.}}$$

The maximum likelihood-estimators of log-linear Poisson models are equal to the estimators obtained from the conditional multinomial likelihood. The cells of a contingency table, representing the capture histories, are assumed to have independent Poisson distributions. As with the multinomial likelihood, probabilities associated with a certain capture history, can be parameterized into probabilities of being captured in a certain sample, $(e.g. p_{1})$. The “Poisson”-approach allows for the flexible modelling of dependencies between samples by the use of interaction terms. A saturated model cannot be fit since the contingency table is incomplete; cell $n_{0}$ is unobserved.

### Direct modelling approach suggested by Jones et al. (Bayesian framework)

Jones et al. demonstrated an alternative modelling approach, based on an interpretable parameterization and driven by careful consideration of the relationships between the sources. They argued that the log-linear approach is too restrictive to model complex interaction such as referrals. They proposed direct modelling of these referrals and chose the Bayesian software WinBUGS for pragmatic reasons. The same models could be fitted in a maximum likelihood framework.

### Chao’s sample coverage, Burnham’s jackknife (Non-parametric framework)

Burnham and Overton developed a non-parametric procedure for the estimation of the size of a closed population. The method allows for heterogeneous, individual, capture probabilities, but assumes these are constant over capture occasions. The method thus assumes equal sampling effort on each occasion. Jackknife resampling is a method in which bias reduction is done by predicting new observations. New observations, $N_{t}^{*}$, also called pseudo-values, are found by subtracting partial estimates $N_{-t}$ (all the cases except those uniquely captured on occasion $t$) from $N_{obs}$ (all captured cases) as follows; let $n$ be the number of occassions

$$N_{t}^{*}=nN_{obs}-\left( n-1 \right)N_{-t}$$

The new estimate, the first order jackknife-estimate is then found by averaging over the predicted values $\frac{\sum_{t}^{\tau} N_{t}^{*}}{n}$. For higher order jackknife-estimates, please see Burnham and Overton’s paper [5].

Chao estimated the population size via the overlap between the population size and the sample coverage. In a three-sample study the sample coverage ($\hat{C}$) is the average of the three possible overlap fractions.

$$\hat{C}=\frac{1}{3}\left[ \frac{\sum_{i=1}^{N} P_{i3}I(X_{i1}+X_{i2} >0)}{\sum_{i=1}^{N} P_{i3}}+\frac{\sum_{i=1}^{N} P_{i2}I(X_{i1}+X_{i3} >0)}{\sum_{i=1}^{N} P_{i2}}+ \frac{\sum_{i=1}^{N} P_{i1}I(X_{i2}+X_{i3} >0)}{\sum_{i=1}^{N} P_{i1}} \right]$$

$P_{i3}$ is the capture probability of case $i$ in sample$3$. $I()$ is the indicator function. $X_{i1}=0$ if case $i$ is not captured in sample 1 and 1 otherwise. If $D$ now represents the average number of cases detected in a sample, but also detected in one or multiple other samples (overlapping cases), then an estimator (in case of independence between sources) would be;

$$\hat{N}=D/\hat{C}$$

In case of dependence between the samples, an adjusted estimator based on a function of the coefficient of covariation is suggested [6].

## Relative bias, standard error and RMSE

The relative bias is positive when the estimate is lower than the actual population size and negative in case of overestimation. The relative bias of the estimators is compared to the relative bias (and the negative of this relative bias) of the estimator obtained by summing up the unique cases. The non-parametric estimators have a larger relative bias in the scenario without heterogeneity compared to the “unique cases”-estimator. In two of the three scenarios with one source of heterogeneity the jackknife-estimator has a larger bias than the “unique cases”-estimator (**Fig 1** and **Fig 2**).


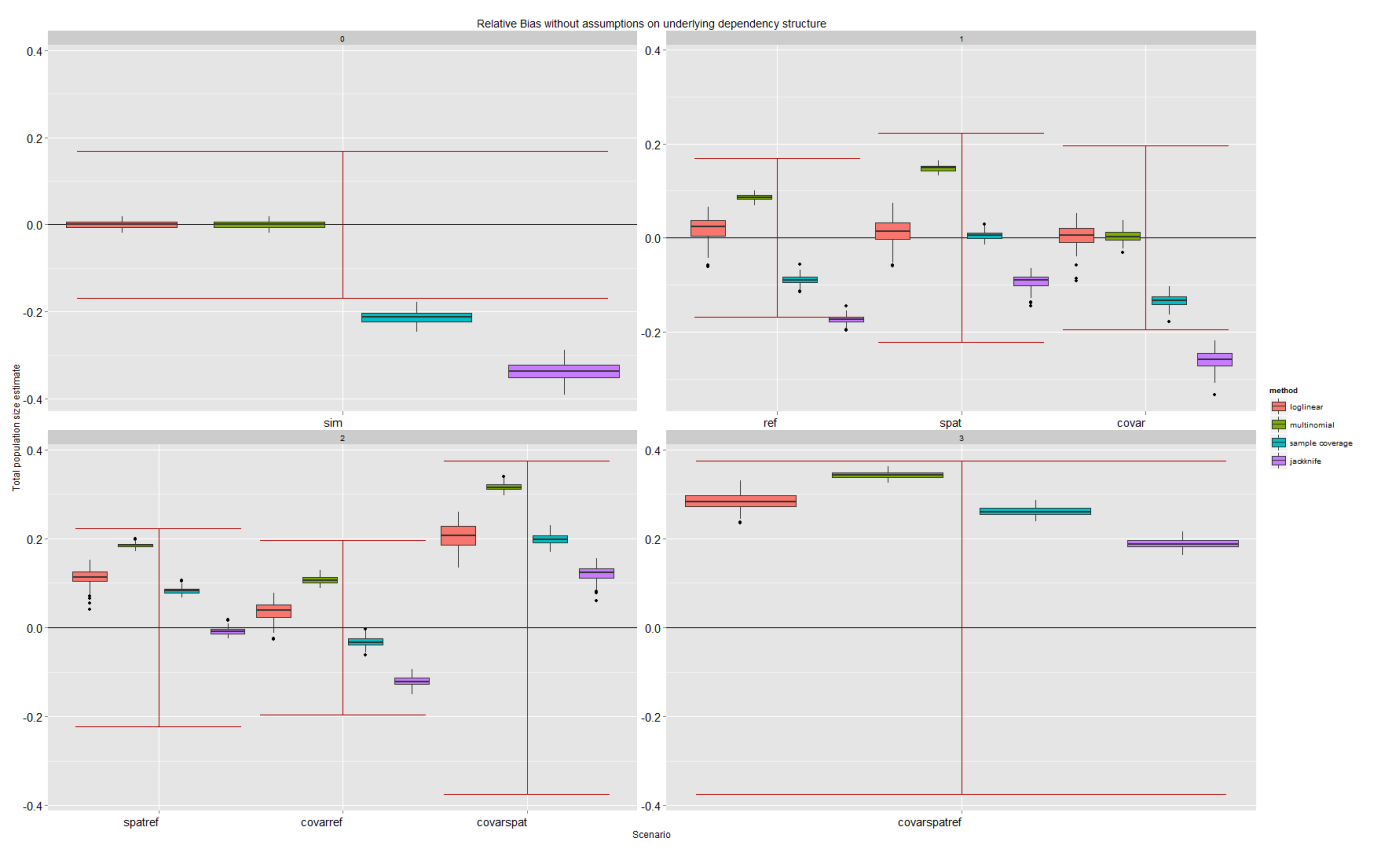


**Fig 1: Boxplots of the relative bias of the obtained estimates per scenario and method. The models were chosen based on AIC, without assumptions on the underlying dependency structure. The black line indicates a relative bias of zero. Presented as red interval is the relative bias (and negative of the relative bias) of the estimator obtained by summing up the unique cases.**


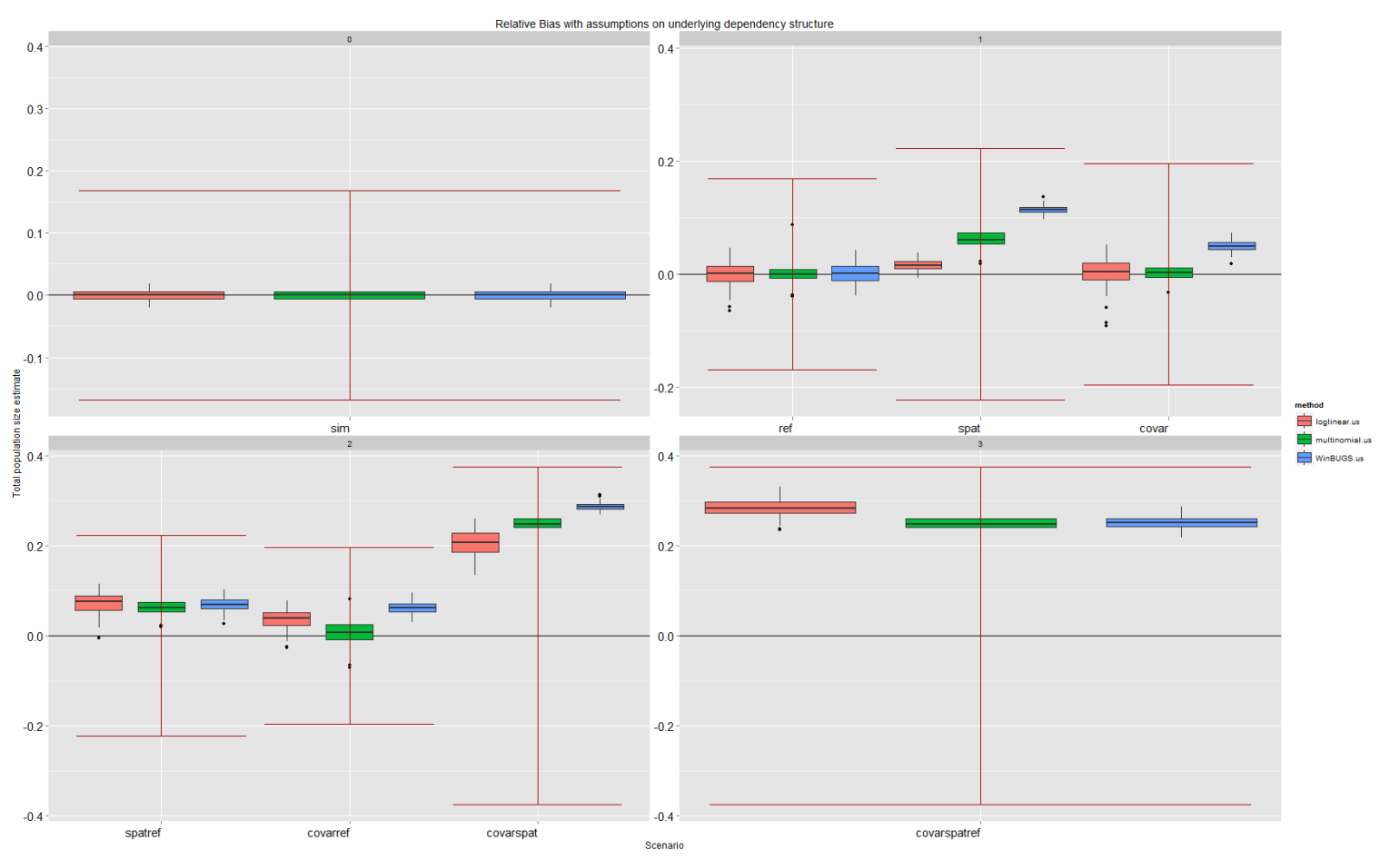


**Fig 2: Boxplots of the relative bias of the obtained estimates per scenario and method. The models were chosen based on assumptions on the underlying dependency structure. The black line indicates a relative bias of zero. Presented as red interval is the relative bias (and negative of the relative bias) of the estimator obtained by summing up the unique cases.**

## WinBUGS-code

See Jones et al. [7]

For the model $\sim p_{1},p_{2},p_{3}s_{11},p_{3}s_{10}, q_{13},q_{23}$

*model{*

*# Split the full multinomial likelihood into two parts*

*# First part: multinomial likelihood conditional on being observed:*

*x[1:7] ~ dmulti(prob.obs[1:7], x.obs)*

*x.obs <- sum(x[1:7])*

*for(i in 1:7){*

*# Probability of being in the ith cell conditional on being observed:*

*prob.obs[i] <- prob[i]/sum(prob[1:7])*

*}*

*# Second part: binomial likelihood for being observed*

*x.obs ~ dbin(sum.prob, N)*

*sum.prob <- sum(prob[1:7]) # probability of being observed*

*xmiss <- N - x.obs # number of missing individuals*

*# Specify the 8 cell probabilities directly*

*# in terms of intuitive parameters:*

*prob[1] <- p2*(p3S11*p1+(q13+q23-q13*q23)*(1-p3S11)*p1)*

*prob[2] <- p1*p2*(1-(p3S11+(q13+q23-q13*q23)*(1-p3S11)))*

*prob[3] <- (1-p2)*(p3S11*p1 + q13*(1-p3S11)*p1)*

*prob[4] <- (p1*(1-p2)*(1-(p3S11+q13*(1-p3S11))))*

*prob[5] <- (1-p1)*p2*(p3S10+q23*(1-p3S10))*

*prob[6] <- (1-p1)*p2*(1-(p3S10+q23*(1-p3S10)))*

*prob[7] <- (1-p1)*(1-p2)*p3S10*

*prob[8] <- (1-p1)*(1-p2)*(1-p3S10)*

*# Vague prior distributions for probabilities:*

*p1 ~dunif(0,1)*

*p2 ~ dunif(0,1)*

*p3S10 ~ dunif(0,1)*

*p3S11 ~ dunif(0,1)*

*# Vague prior for proportion referred from Source 3 into Source 1:*

*q13 ~ dunif(0,1)*

*q23 ~ dunif(0,1)*

*# Vague prior for total population size:*

*log.x.obs <- log(x.obs)*

*log(N) <- logN*

*logN ~ dnorm(0, 0.0001)I(log.x.obs,)*

*}*

## References

1. Otis DL, Burnham KP, White GC, Anderson DR. Statistical Inference from Capture Data on Closed Animal Populations. Wildl Monogr. 1978;(62):3–135.

2. Darroch JN. The Multiple-Recapture Census: I. Estimation of a Closed Population. Biometrika. 1958 Dec;45(3/4):343.

3. Sanathanan L. Estimating the Size of a Multinomial Population. Ann Math Stat. 1972 Feb;43(1):142–52.

4. Horvitz DG, Thompson DJ. A Generalization of Sampling Without Replacement From a Finite Universe. J Am Stat Assoc. 1952 Dec;47(260):663.

5. Burnham KP, Overton WS. Estimation of the size of a closed population when capture probabilities vary among animals. Biometrika. 1978 Dec 1;65(3):625–33.

6. Chao A, Tsay PK. A Sample Coverage Approach to Multiple-System Estimation with Application to Census Undercount. J Am Stat Assoc. 1998;93(441):283–93.

7. Jones HE, Hickman M, Welton NJ, De Angelis D, Harris RJ, Ades AE. Recapture or Precapture? Fallibility of Standard Capture-Recapture Methods in the Presence of Referrals Between Sources. Am J Epidemiol. 2014 Apr 11;
